# Supplementary material for: Decoding the activated stem cell phenotype of the neonatally maturing pituitary
Source: eLife. 2022 Jun 14;11:e75742. doi: 10.7554/eLife.75742 (PMC9333987; doi:10.7554/eLife.75742)
Supplement: Transparent reporting form [file elife-75742-transrepform1.docx]

**eLife’s transparent reporting form**

We encourage authors to provide detailed information *within their submission* to facilitate the interpretation and replication of experiments. Authors can upload supporting documentation to indicate the use of appropriate reporting guidelines for health-related research (see [EQUATOR Network](http://www.equator-network.org/%20)), life science research (see the [BioSharing Information Resource](https://biosharing.org/)), or the [ARRIVE guidelines](http://www.plosbiology.org/article/info:doi/10.1371/journal.pbio.1000412) for reporting work involving animal research. Where applicable, authors should refer to any relevant reporting standards documents in this form.

If you have any questions, please consult our Journal Policies and/or contact us: [editorial@elifesciences.org](mailto:editorial@elifesciences.org).

**Sample-size estimation**

- You should state whether an appropriate sample size was computed when the study was being designed
- You should state the statistical method of sample size computation and any required assumptions
- If no explicit power analysis was used, you should describe how you decided what sample (replicate) size (number) to use

Please outline where this information can be found within the submission (e.g., sections or figure legends), or explain why this information doesn’t apply to your submission:

No explicit power analyses were used. We performed the appropriate number of biological replicates per research question (for both mouse and organoid experiments; see below), in accordance to prevailing biological research standards as well as based on our extensive prior experience and expertise when designing these type of experiments. Sample sizes are clear from figures and figure legends.

**Replicates**

- You should report how often each experiment was performed
- You should include a definition of biological versus technical replication
- The data obtained should be provided and sufficient information should be provided to indicate the number of independent biological and/or technical replicates
- If you encountered any outliers, you should describe how these were handled
- Criteria for exclusion/inclusion of data should be clearly stated
- High-throughput sequence data should be uploaded before submission, with a private link for reviewers provided (these are available from both GEO and ArrayExpress)

Please outline where this information can be found within the submission (e.g., sections or figure legends), or explain why this information doesn’t apply to your submission:

The number of biological replicates is indicated in most figures by the number of individual dots and/or stated in the figure legends. Each experiment was independently repeated at least 3 times (biological replication), unless otherwise stated in the Results or Methods section. The number of technical replicates (i.e. replication within a single biological experiment) can be found in the figure legends where relevant.

The single cell RNA-sequencing (scRNA-seq) dataset can be found on ArrayExpress (accession number E-MTAB-11337), as also mentioned in the Methods section. Quality control steps in the scRNA-seq analysis, aimed at excluding potential doublets and low-quality cells, followed standard workflows and are described in the Methods section (i.e. cells with less than 750 genes or more than 8,000 genes or more than 17.5% mitochondrial RNA were removed).

**Statistical reporting**

- Statistical analysis methods should be described and justified
- Raw data should be presented in figures whenever informative to do so (typically when N per group is less than 10)
- For each experiment, you should identify the statistical tests used, exact values of N, definitions of center, methods of multiple test correction, and dispersion and precision measures (e.g., mean, median, SD, SEM, confidence intervals; and, for the major substantive results, a measure of effect size (e.g., Pearson's r, Cohen's d)
- Report exact p-values wherever possible alongside the summary statistics and 95% confidence intervals. These should be reported for all key questions and not only when the p-value is less than 0.05.

Please outline where this information can be found within the submission (e.g., sections or figure legends), or explain why this information doesn’t apply to your submission:

Statistical analysis was performed as explained in the Methods section. Figure legends define the data shown in the figure (usually mean ± SEM, unless otherwise stated) and the used statistical tests. Individual data points and exact p-values are presented in most figures.

(For large datasets, or papers with a very large number of statistical tests, you may upload a single table file with tests, Ns, etc., with reference to sections in the manuscript.)

**Group allocation**

- Indicate how samples were allocated into experimental groups (in the case of clinical studies, please specify allocation to treatment method); if randomization was used, please also state if restricted randomization was applied
- Indicate if masking was used during group allocation, data collection and/or data analysis

Please outline where this information can be found within the submission (e.g., sections or figure legends), or explain why this information doesn’t apply to your submission:

Samples were either randomly allocated to experimental groups (i.e. for treatments of mice or organoid cultures), or where allocated according to their genotype (i.e. for comparison of pituitary stem cell activation and organoids from IL-6 knock-out *versus* wildtype mice and for regeneration analysis using a transgenic damage mouse model).

**Additional data files (“source data”)**

- We encourage you to upload relevant additional data files, such as numerical data that are represented as a graph in a figure, or as a summary table
- Where provided, these should be in the most useful format, and they can be uploaded as “Source data” files linked to a main figure or table
- Include model definition files including the full list of parameters used
- Include code used for data analysis (e.g., R, MatLab)
- Avoid stating that data files are “available upon request”

Please indicate the figures or tables for which source data files have been provided:

‘Figure 1 – figure supplement 2A’ has associated source data file ‘Figure 1 – figure supplement 2 - Source Data 1’.

‘Figure 1 – figure supplement 2B’ has associated source data file ‘Figure 1 – figure supplement 2 - Source Data 2’.

‘Figure 1 – figure supplement 2C’ has associated source data file ‘Figure 1 – figure supplement 2 - Source Data 3’.

‘Figure 3A and B’ has associated source data file ‘Figure 3 – Source Data 1’.

‘Figure 4 – figure supplement 2A’ has associated source data file ‘Figure 4 – figure supplement 2 - Source Data 1’.

‘Figure 4 – figure supplement 2B’ has associated source data file ‘Figure 4 – figure supplement 2 - Source Data 2’.

This information is indicated in the Results.
